# Lack of associations between *AURKA* gene polymorphisms and neuroblastoma susceptibility in Chinese children

**DOI:** 10.1042/BSR20180292

**Published:** 2018-05-22

**Authors:** Jue Tang, Yuanmin Qian, Jinhong Zhu, Jiao Zhang, Feng-Hua Wang, Jia-Hang Zeng, Jiang-Hua Liang, Hui Wang, Huimin Xia, Jing He, Wei Liu

**Affiliations:** 1 Department of Pediatric Surgery, Guangzhou Institute of Pediatrics, Guangzhou Women and Children’s Medical Center, Guangzhou Medical University, Guangzhou 510623, Guangdong, China; 2 Department of Gynecology and Obstetrics, Guangzhou Women and Children’s Medical Center, Guangzhou Medical University, Guangzhou 510623, Guangdong, China; 3 Department of Clinical Laboratory, Molecular Epidemiology Laboratory, Harbin Medical University Cancer Hospital, Harbin 150040, Heilongjiang, China; 4 Department of Pediatric Surgery, The First Affiliated Hospital of Zhengzhou University, Zhengzhou 450052, Henan, China

**Keywords:** AURKA, neuroblastoma, polymorphism, susceptibility

## Abstract

Previous studies have demonstrated that polymorphisms in the *AURKA* gene are associated with various types of cancer. In neuroblastoma, *AURKA* protein product regulates N-myc protein levels and plays a critical role in tumorigenesis. To investigate the association between three *AURKA* polymorphisms (rs1047972 C>T, rs2273535 T>A, and rs8173 G>C) and neuroblastoma susceptibility in Chinese populations, we performed this two-center case–control study including 393 neuroblastoma cases and 812 controls. Two study populations were recruited from two different regions in China. No significant associations were identified amongst any of the three *AURKA* polymorphisms and the risk of neuroblastoma. Similar observations were found in the stratified analysis. In conclusion, our results indicate that none of the *AURKA* polymorphisms are associated with neuroblastoma susceptibility in two distinct Chinese populations. Further studies with larger sample sizes and different ethnicities are warranted to validate our results.

## Introduction

Neuroblastoma is a neuroendocrine tumor that originates from the developing sympathetic nervous system. It is the most common solid malignancy in the first year of life, accounting for approximately 15% of pediatric cancer deaths [1]. Despite remarkable advances in multimodality treatment, the survival rate for patients with high-risk tumors remains approximately 50% [1,2]. At present, the etiology of neuroblastoma remains far from clear. Genetic variations have been shown to be important factors in the origination and development of neuroblastoma [3–5]. A previous genome-wide association study (GWAS) demonstrated that common genetic variations in the *BARD1* gene may contribute to the etiology of the aggressive neuroblastoma [6]. Moreover, polymorphisms in the *CDKN1B* gene were found to associate with neuroblastoma susceptibility [7]. Over the past years, several GWASs have identified a number of genetic alterations that not only influence neuroblastoma formation but also contribute to malignant transformation [8–10]. Single nucleotide polymorphisms (SNPs) within *DDX4, HSD17B12*, and *DUSP12* are recurrent in low-risk neuroblastoma [11,12]. SNPs in *CASC15, LMO1*, and *LIN28B* are enriched in high-risk neuroblastoma and are correlated with neuroblastoma tumor aggressiveness [10,13,14]. Previous study demonstrated that LIN28B promotes *AURKA* expression via inhibition of let-7, further driving neuroblastoma oncogenesis [15].

*AURKA*, located at chromosome region 20q13.2, encodes a serine/threonine kinase (Aurora-A) that has been shown to play a crucial role in regulating mitosis. Overexpression of Aurora-A contributes to centrosomal duplication abnormalities, genomic instability, and the promotion of tumorigenesis [16,17]. Over the past decade, Aurora-A overexpression has been demonstrated in multiple human cancers, including primary colorectal carcinoma, esophageal squamous cell carcinoma, and breast and ovarian cancers [17–19]. And overexpression of Aurora-A is associated with advanced clinical states, worse overall survival, and shorter event-free survival in patients with neuroblastoma [20]. In addition, several studies confirmed that *AURKA* polymorphisms were associated with the risk of several human cancers [21–24]. To date, no study has assessed the associations between *AURKA* SNPs and the risk of neuroblastoma.

To evaluate the associations between the SNPs in the *AURKA* gene and neuroblastoma susceptibility, we conducted this case–control study with 393 neuroblastoma cases and 812 control subjects from the Chinese population.

## Materials and methods

### Study subjects

In the present study, a total of 393 neuroblastoma cases and 812 controls were recruited from two different regions of China [25–28]. The first population was composed of 275 neuroblastoma cases and 531 controls from the Guangzhou Women and Children’s Medical Center [29–31]. The second population consisted of 118 neuroblastoma cases and 281 controls from The First Affiliated Hospital of Zhengzhou University [32,33]. Both the cases and controls were of Chinese Han ethnicity and were genetically unrelated. The cases and controls were matched according to age, gender, and ethnicity. Neuroblastoma patients were diagnosed by biopsy and staged according to the International Neuroblastoma Staging System (INSS) [34]. The present study was approved by the Institutional Review Board of each institution. Written informed consent was acquired from the parents or guardians of each participant.

### Polymorphism selection and genotyping

Polymorphisms were chosen based on the following criteria: (i) minor allele frequency >5% for CHB subjects; (ii) potentially functional as predicted by SNPinfo (http://snpinfo.niehs.nih.gov/) software; (iii) not investigated for the association with neuroblastoma susceptibility. We searched the potentially functional polymorphisms located in the 5′-flanking region, 5′ UTR, 3′ UTR, and exon of *AURKA* gene*.* Three SNPs (rs1047972 C>T, rs2273535 T>A, and rs8173 G>C) in the *AURKA* gene were selected (Supplementary Table S1). These three SNPs can capture additional fourteen SNPs. As shown in Supplementary Figure S1, there was no significant linkage disequilibrium between paired polymorphisms (*R^2^* = 0.119 between rs8173 and rs1047972; *R^2^* = 0.527 between rs8173 and rs2273535; and the *R^2^* = 0.291 between rs1047972 and rs2273535). These SNPs were genotyped using TaqMan real-time PCR following a published protocol [35–38]. To ensure credible genotyping results, 10% of the samples were randomly selected for repeated genotyping assays, and the results were 100% concordant.

### Statistical analysis

Differences in genotype frequencies as well as in demographic variables between cases and controls were compared by two-sided χ^2^ tests. Hardy–Weinberg equilibrium (HWE) for the genotype frequencies in controls was calculated by a goodness-of-fit χ^2^ test. Associations between *AURKA* SNPs and neuroblastoma were estimated using adjusted odds ratios (ORs) and 95% confidence intervals (CIs). We also performed analyses stratified by age, gender, tumor sites, and clinical stages. *P*<0.05 was considered statistically significant. All statistical tests were performed using SAS software (version 9.4; SAS Institute, Cary, NC, U.S.A.).

## Results

### AURKA gene polymorphisms and neuroblastoma susceptibility

In the present study, 393 cases and 812 controls were successfully genotyped. The genotype frequencies of the three *AURKA* polymorphisms and their associations with neuroblastoma susceptibility are summarized in Table 1. The observed genotype frequencies amongst the control subjects were in HWE (*P*=0.337 for the rs1047972 C>T polymorphism, *P*=0.174 for the rs2273535 T>A polymorphism, and *P*=0.506 for the rs8173 G>C polymorphism). No significant associations were identified between any of the three *AURKA* SNPs and the risk of neuroblastoma.

**Table 1:** The correlation of *AURKA* gene polymorphisms with neuroblastoma risk

Genotype	Cases (*n*=393)	Controls (*n*=812)	*P* ^1^	Crude OR (95% CI)	*P*	Adjusted OR (95% CI)^2^	*P* ^2^
**rs1047972 C>T (HWE = 0.337)**							
CC	305 (77.61)	629 (77.46)		1.00		1.00	
CT	87 (22.14)	168 (20.69)		1.07 (0.80–1.43)	0.660	1.07 (0.80–1.43)	0.671
TT	1 (0.25)	15 (1.85)		0.14 (0.02–1.05)	0.055	0.14 (0.02–1.04)	0.055
**Additive**			0.070	0.92 (0.70–1.20)	0.535	0.92 (0.70–1.20)	0.526
Dominant	88 (22.39)	183 (22.54)	0.955	0.99 (0.74–1.32)	0.955	0.99 (0.74–1.32)	0.943
Recessive	392 (99.75)	797 (98.15)	0.024	0.14 (0.02–1.03)	0.053	0.14 (0.02–1.03)	0.053
**rs2273535 T>A (HWE = 0.174)**							
TT	182 (46.31)	377 (46.43)		1.00		1.00	
TA	171 (43.51)	340 (41.87)		1.04 (0.81–1.35)	0.753	1.04 (0.81–1.34)	0.765
AA	40 (10.18)	95 (11.70)		0.87 (0.58–1.31)	0.513	0.87 (0.58–1.31)	0.511
**Additive**			0.699	0.97 (0.81–1.16)	0.735	0.97 (0.81–1.16)	0.728
Dominant	211 (53.69)	435 (53.57)	0.969	1.01 (0.79–1.28)	0.969	1.00 (0.79–1.28)	0.981
Recessive	353 (89.82)	717 (88.30)	0.433	0.86 (0.58–1.26)	0.433	0.86 (0.58–1.27)	0.435
**rs8173 G>C (HWE = 0.506)**							
GG	164 (41.73)	314 (38.67)		1.00		1.00	
GC	176 (44.78)	389 (47.91)		0.87 (0.67–1.12)	0.278	0.86 (0.67–1.12)	0.265
CC	53 (13.49)	109 (13.42)		0.93 (0.64–1.36)	0.711	0.93 (0.64–1.36)	0.698
**Additive**			0.555	0.94 (0.78–1.12)	0.473	0.94 (0.78–1.12)	0.458
Dominant	229 (58.27)	498 (61.33)	0.309	0.88 (0.69–1.13)	0.309	0.88 (0.69–1.12)	0.294
Recessive	340 (86.51)	703 (86.58)	0.976	1.01 (0.71–1.43)	0.976	1.00 (0.71–1.43)	0.981
**Combined effect of protective genotypes**^3^							
0	162 (41.22)	312 (38.42)		1.00		1.00	
1–3	231 (58.78)	500 (61.58)	0.351	0.89 (0.70–1.14)	0.351	0.89 (0.69–1.13)	0.335

1 *χ^2^* test for genotype distributions between neuroblastoma patients and cancer-free controls.

2 Adjusted for age and gender.

3 Protective genotypes were rs1047972 TT, rs2273535 AA, and rs8173 GC/CC.

### Stratification analysis

We then divided participants into subgroups according to age, gender, clinical stage, and site of origin. The effects of the selected polymorphisms on the risk of neuroblastoma were assessed in this stratified analysis (Table 2). The effects of combined risk genotypes on neuroblastoma risk were also assessed. However, no significant association was discovered for any of the selected polymorphisms.

**Table 2:** Stratification analysis for association between *AURKA* gene genotypes and neuroblastoma susceptibility

Variables	rs1047972 (case/control)		AOR (95% CI)	*P* ^1^	rs2273535 (case/control)		AOR (95% CI)	*P* ^1^	rs8173 (case/control)		AOR (95% CI)	*P*^1^	Protective genotypes (case/control)		AOR (95% CI)	*P*^1^
	CC	CT/TT			TT	TA/AA			GG	GC/CC			0	1–3		
**Age, months**																
≤18	103/241	23/64	0.84 (0.50–1.43)	0.522	64/147	62/158	0.90 (0.60–1.37)	0.624	59/132	67/173	0.87 (0.57–1.32)	0.501	59/130	67/175	0.84 (0.56–1.28)	0.425
>18	202/388	65/119	1.05 (0.74–1.48)	0.789	118/230	149/277	1.05 (0.78–1.41)	0.759	105/182	162/325	0.86 (0.64–1.17)	0.344	103/182	164/325	0.89 (0.66–1.21)	0.458
**Gender**																
Female	130/269	38/73	1.09 (0.70–1.70)	0.715	77/161	91/181	1.06 (0.73–1.53)	0.773	67/139	101/203	1.05 (0.72–1.53)	0.821	66/138	102/204	1.06 (0.72–1.55)	0.772
Male	175/360	50/110	0.93 (0.64–1.36)	0.711	105/216	120/254	0.97 (0.70–1.33)	0.834	97/175	128/295	0.78 (0.56–1.07)	0.123	96/174	129/296	0.78 (0.57–1.08)	0.136
**Sites of origin**																
Adrenal gland	118/629	35/183	1.00 (0.66–1.51)	0.999	67/377	86/435	1.09 (0.77–1.55)	0.623	56/314	97/498	1.06 (0.74–1.51)	0.768	55/312	98/500	1.08 (0.75–1.54)	0.696
Retroperitoneal	68/629	19/183	0.96 (0.57–1.65)	0.894	40/377	47/435	1.02 (0.75–1.59)	0.941	38/314	49/498	0.82 (0.52–1.28)	0.381	37/312	50/500	0.85 (0.54–1.33)	0.476
Mediastinum	87/629	22/183	0.88 (0.54–1.45)	0.621	55/377	54/435	0.86 (0.58–1.28)	0.460	52/314	57/498	0.71 (0.47–1.06)	0.090	52/312	57/500	0.70 (0.47–1.04)	0.080
Others	27/629	9/183	1.14 (0.53–2.47)	0.737	17/377	19/435	0.97 (0.50–1.89)	0.922	15/314	21/498	0.90 (0.45–1.77)	0.750	15/312	21/500	0.88 (0.45–1.75)	0.723
**Clinical stage**																
I + II + 4s	131/629	31/183	0.82 (0.54–1.26)	0.367	80/377	82/435	0.90 (0.64–1.26)	0.520	77/314	85/498	0.71 (0.50–0.996)	0.047	75/312	87/500	0.74 (0.52–1.04)	0.079
III + IV	158/629	53/183	1.14 (0.80–1.62)	0.466	93/377	118/435	1.09 (0.80–1.48)	0.573	77/314	134/498	1.07 (0.78–1.47)	0.660	77/312	134/500	1.06 (0.78–1.46)	0.699

Abbreviation: AOR, adjusted OR.

1 Adjusted for age and gender, omitting the corresponding stratify factor.

## Discussion

We conducted the present case–control study with a total of 393 neuroblastoma patients and 812 control subjects to investigate the impact of three *AURKA* SNPs on the risk of neuroblastoma in Chinese populations. Our data indicated that none of the selected SNPs were associated with neuroblastoma susceptibility in two independent Chinese populations. To the best of our knowledge, this is the first study to investigate the association between neuroblastoma susceptibility and polymorphisms in the *AURKA* gene.

*AURKA* has been reported to be overexpressed in several human malignancies and encodes a serine/threonine kinase that is involved in the processes of proliferation, survival, invasion, and stemness in multiple types of cancer [17]. Several studies have demonstrated that *AURKA* SNPs were associated with the risk of cancer [21–23]. Lee at al. [39] identified an association between a genetic variant (rs2273535) in the *AURKA* gene and oral cancer. A recent study has demonstrated that *AURKA* SNPs (rs1047972 and rs2273535) increase the risk of oral squamous cell carcinoma [40]. In Malaysian Chinese, *AURKA* rs2273535 protected against breast cancer [41]. A meta-analysis suggested that *AURKA* rs1047972 is associated with a decreased breast cancer risk in Caucasians, while *AURKA* rs2273535 polymorphism is associated with an increased risk of breast cancer [23]. This finding indicates that the functions of *AURKA* SNPs may vary depending on the types of cancer and ethnic differences.

Aurora A is responsible for stabilizating N-myc in neuroblastoma. [17,42]. A previous study demonstrated that LIN28B-RAN-AURKA axis is implicated in neuroblastoma oncogenesis. Aurora A overexpression in neuroblastoma is associated with advanced clinical states, *MYCN* amplification, disease relapse, and progression [43]. As a transcriptional regulator, *MYCN* (encoding N-myc) plays a crucial role during embryonic development. In addition, *MYCN* amplification, which is involved in the inhibition of both cell-cycle exit and normal differentiation, contributes to neuroblastoma initiation and progression [44–46]. Knockdown of *AURKA* has been shown to decrease N-myc protein levels and neuroblastoma cell proliferation [47]. However, we failed to detect any significant association between these *AURKA* SNPs (rs1047972 C>T, rs2273535 T>A, and rs8173 G>C) and neuroblastoma susceptibility in the present study. The negative results might be attributed to the limited sample size. The relatively small sample size might not be large enough to detect an association.

Several limitations in our study should be mentioned. First, the sample size in the present study might not be large enough to draw accurate conclusions. Increasing the sample size would increase the power to detect risk variants and increase the credibility of any observed associations. Analyses with large sample sizes are essential to verify our results. Second, the etiology of neuroblastoma is complex and multifactorial. Several important factors such as dietary intake and living environment contribute to neuroblastoma pathogenesis. The results should be explained with caution, because these confounding factors were not included in the current study. Third, only three *AURKA* SNPs were investigated in our study. Other polymorphisms in the *AURKA* gene should be investigated in future study. Fourth, the genotype distribution in this hospital-based study may not reflect that in the general population, which would inevitably result in selection bias.

In summary, our study confirmed that none of the *AURKA* polymorphisms (rs1047972 C>T, rs2273535 T>A, and rs8173 G>C) were associated with neuroblastoma susceptibility in two distinct Chinese populations. Future studies with larger sample sizes and different ethnicities are required to further clarify the effect of *AURKA* SNPs on the risk of neuroblastoma.

## Supporting information

**Supplemental 1:** Linkage disequilibrium analysis for the three selected. *AURKA* polymorphisms in Han Chinese population consisted of CHB (Han Chinese in Beijing, China) and CHS (Southern Han Chinese) subjects.

**Supplemental Table S1:** Polymorphism captured by the three selected *AURKA* potentially funnction polymorphisms as predicted by SPNinfo online software (https://snpinfo.niehs.nih.gov/snpinfo/snpfunc.html)

## Abbreviations

GWAS: genome-wide association study
HWE: Hardy–Weinberg equilibrium
SNP: single nucleotide polymorphism
