# Supplementary material for: Lack of associations between AURKA gene polymorphisms and neuroblastoma susceptibility in Chinese children
Source: Biosci Rep. 2018 May 22;38(3):BSR20180292. doi: 10.1042/BSR20180292 (PMC6048213; doi:10.1042/BSR20180292)
Supplement: Supplementary file 1 [file bsr20180292_Supp1.pdf]

Supplementary Information

Supplemental Table 1. Polymorphisms captured by the three selected *AURKA* potentially functional polymorphisms as predicted by SNPinfo online software (https://snpinfo.niehs.nih.gov/snpinfo/snpfunc.html)

rs	Chr.	Allele	LDsnp	Pop/LD	TFBS	Splicing (ESE or ESS)	miRNA (miRanda)	nsSNP	Allele	Asian	CHB
**rs1047972**	**20**	**T/C**	**rs1047972**	**1**	**--**	**--**	**--**	**Y**	**C**	**0.861**	**--**
rs2180691	20	A/G	rs2273535	CHB/0.815	Y	--	--	--	A	0.661	0.667
**rs2273535**	**20**	**A/T**	**rs2273535**	**1**	**--**	**Y**	**--**	**Y**	**A**	**0.797**	**0.367**
rs2298016	20	C/G	rs2273535	CHB/1	--	--	--	--	G	0.367	0.367
rs6024840	20	A/G	rs2273535	CHB/0.845	--	--	--	--	A	0.325	0.369
rs6127737	20	A/G	rs2273535	CHB/0.904	Y	--	--	--	G	0.356	0.369
rs911160	20	C/G	rs2273535	CHB/0.904	--	--	--	--	C	0.677	0.649
rs1044377	20	G/T	rs8173	CHB/0.953	--	--	Y	--	T	0.622	0.600
rs11698420	20	G/T	rs8173	CHB/0.807	--	--	--	--	G	0.360	0.364
rs1926071	20	G/T	rs8173	CHB/0.811	--	--	--	--	G	0.624	0.633
rs1926074	20	A/G	rs8173	CHB/0.811	--	--	--	--	G	0.633	0.622
rs2209593	20	C/T	rs8173	CHB/0.811	--	--	--	--	C	0.619	0.622
rs4811692	20	A/G	rs8173	CHB/0.808	--	--	--	--	G	0.386	0.411
rs6024833	20	A/G	rs8173	CHB/0.906	--	--	--	--	A	0.378	0.378
rs6024836	20	A/G	rs8173	CHB/0.927	--	--	--	--	A	--	0.583
rs6127729	20	C/T	rs8173	CHB/0.804	--	--	--	--	C	0.369	0.384
**rs8173**	**20**	**C/G**	**rs8173**	**1**	**--**	**--**	**Y**	**--**	**G**	**0.657**	**0.578**

LD, linkage disequilibrium; TFBS, transcription factor binding sites; ESE, exonic splicing enhancer; ESS, exonic splicing silencer; SNP, single nucleotide polymorphism; CHB, Han Chinese in Beijing, China.

Supplemental Figure 1

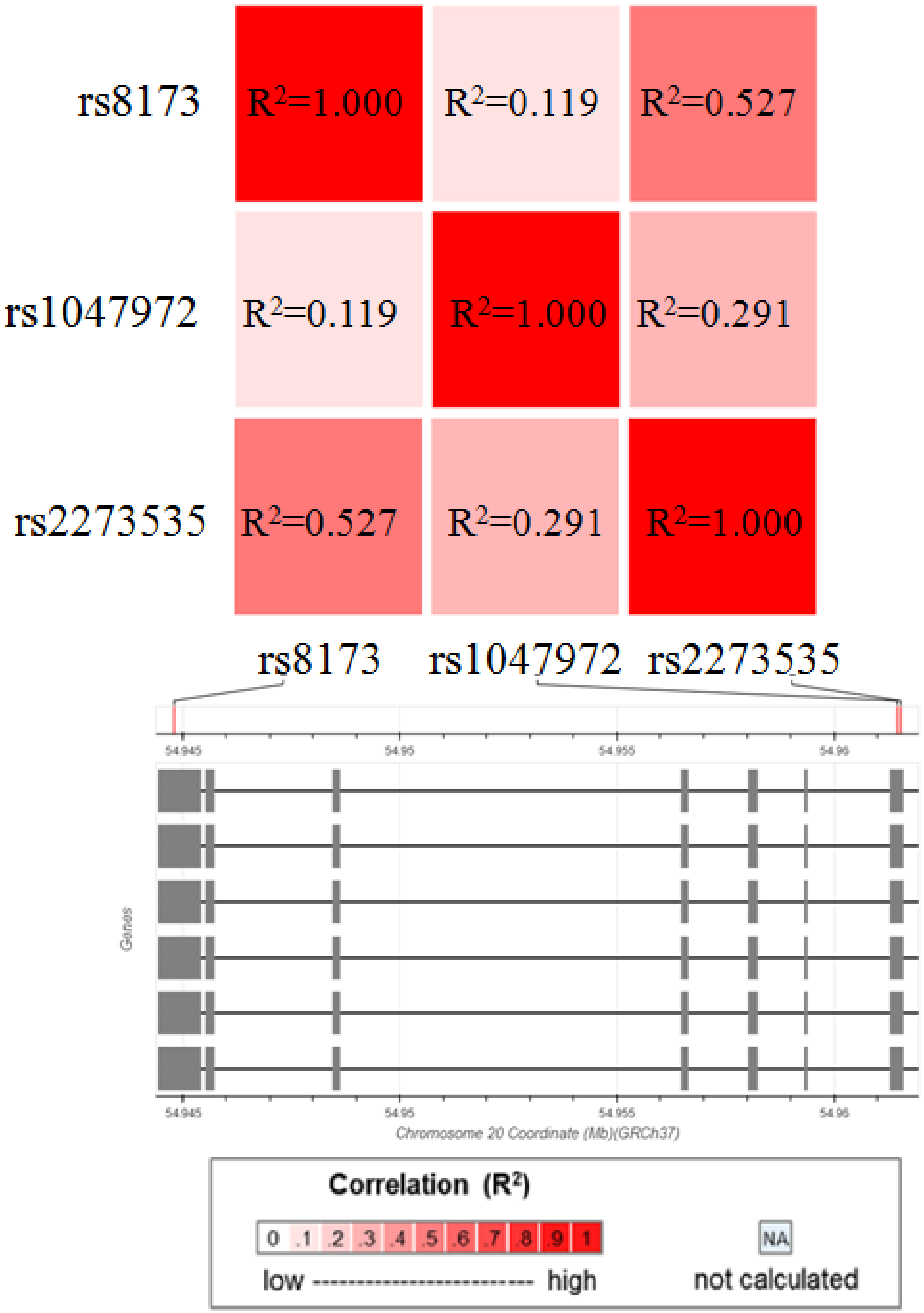

Supplemental Figure 1. Linkage disequilibrium analysis for the three selected *AURKA* polymorphisms in Han Chinese population consisted of CHB (Han Chinese in Beijing, China) and CHS (Southern Han Chinese) subjects.
